# Successful multidisciplinary treatment for synchronous advanced esophageal and cecal cancers after total gastrectomy with reconstruction by jejunal interposition

**DOI:** 10.1186/s12957-024-03361-1

**Published:** 2024-03-14

**Authors:** Yuta Sato, Yoshihiro Tanaka, Kazuo Yamamoto, Takeshi Horaguchi, Masahiro Fukada, Yuki Sengoku, Itaru Yasufuku, Ryuichi Asai, Jesse Yu Tajima, Shigeru Kiyama, Takazumi Kato, Katsutoshi Murase, Nobuhisa Matsuhashi

**Affiliations:** Department of Gastroenterological Surgery and Pediatric Surgery, Gifu University Graduate School of Medicine, 1-1 Yanagido, Gifu Prefecture, Gifu City, 501-1194 Japan

**Keywords:** Esophageal cancer, Colorectal cancer, Synchronous cancers, Reconstruction, Jejunal interposition

## Abstract

**Background:**

Esophageal squamous cell carcinoma is characterized by field cancerization, wherein multiple cancers occur in the esophagus, head and neck, and stomach. Synchronous esophageal and colorectal cancers are also encountered with a certain frequency. A good prognosis can be expected if the tumors in both locations can be safely and completely removed. For patients with multiple cancers that occur simultaneously with esophageal cancer, it is necessary to perform a staged operation, taking into consideration the associated surgical invasiveness. It is also necessary to select multidisciplinary treatment depending on the degree of progression of the multiple lesions. We report our rare experience with a staged operation for a patient with synchronous advanced cancers of the esophagus and cecum who had previously undergone total gastrectomy with reconstruction by jejunal interposition for gastric cancer.

**Case presentation:**

A 71-year-old man with a history of reconstruction by jejunal interposition after total gastrectomy was diagnosed as having multiple synchronous esophageal and cecal cancers. After neoadjuvant chemotherapy, we performed a planned two-stage operation, with esophagectomy and jejunostomy in the first stage and ileocecal resection and jejunal reconstruction with vascular anastomosis in the second. Postoperatively, the patient was relieved without major complications, and both tumors were amenable to curative pathologic resection.

**Conclusions:**

Our procedure reported here may be recommended as an option for staged resection and reconstruction in patients with simultaneous advanced esophageal and cecal cancer after total gastrectomy.

## Background

Patients with a history of cancer may develop several cancers in their lives [1]. When a patient is diagnosed as having more than one cancer, synchronous or metachronous multiple primary cancers may be reported. Recently, the prevalence of multiple primary cancers has risen [2]. The incidence of multiple primary cancers in patients with esophageal cancer is reportedly 5–36% [3–5]. The most frequently occurring multiple primary cancers are gastric, head and neck, and lung cancer, which is characterized by field cancerization [6]. The frequency of synchronous esophageal and colorectal cancers has been reported to be around 2.9–5.8%. [7–9]. Yoshida et al. reported that esophageal cancer patients over 70 years of age with a history of heavy smoking are an independent risk factor for the development of synchronous colorectal cancer [10]. Therefore, although relatively rare, a certain percentage of cases require simultaneous resection of the esophagus and colorectum.

Because esophageal cancer surgery is highly invasive, synchronous resection of both esophageal and multiple primary cancers is controversial. Several reviews indicate that simultaneous resection of esophageal cancer and multiple primary cancers can be safely performed and that complete tumor removal of both tumors is necessary to achieve good long-term results [11, 12]. However, esophageal cancer usually occurs in elderly patients who often have coexisting disease, and these co-morbidities can impair the tolerance of patients to the invasiveness of esophageal surgery. A two-stage operation was particularly applied for high-risk patients in the late twentieth century [13, 14]. Even in recent years, the effectiveness of a staged operation has been reported in high-risk cases, such as patients with esophageal cancer after gastrectomy or those with hepatic or respiratory complications [15–18].

Herein, we report the successful performance of a two-stage operation including oncological multidisciplinary treatment of advanced esophageal and cecal cancer occurring simultaneously in a patient who had previously undergone total gastrectomy with reconstruction using jejunal interposition for gastric cancer.

## Case presentation

**Fig. 1:**
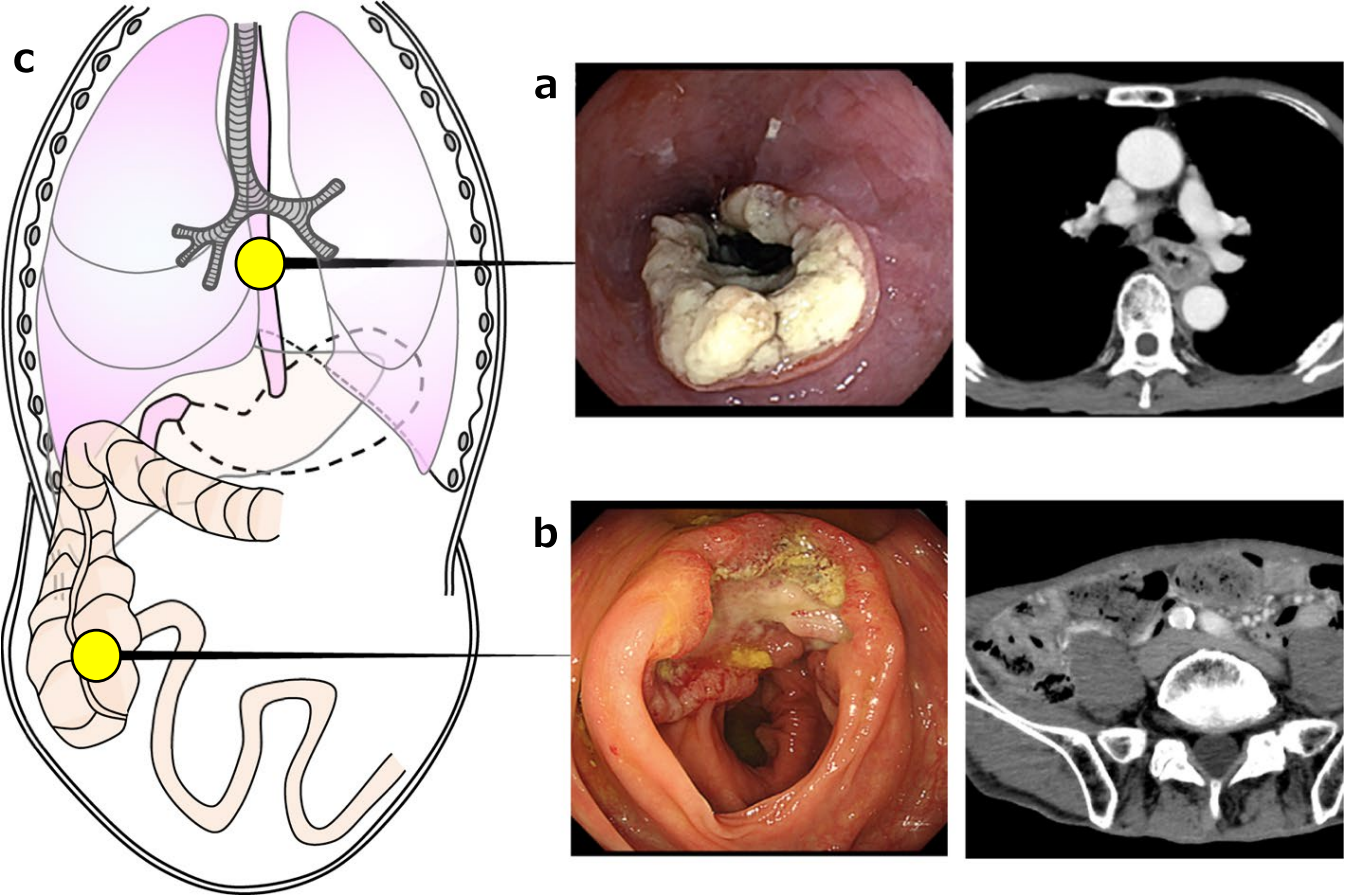
Preoperative endoscopy and contrast-enhanced computed tomography findings. **a** Esophageal squamous cell carcinoma T3N1M0 in the median thoracic esophagus. **b** Cecal cancer T3N0M0. **c** Prior to the start of treatment, the method of reconstruction after total gastrectomy was unknown

**Fig. 2:**
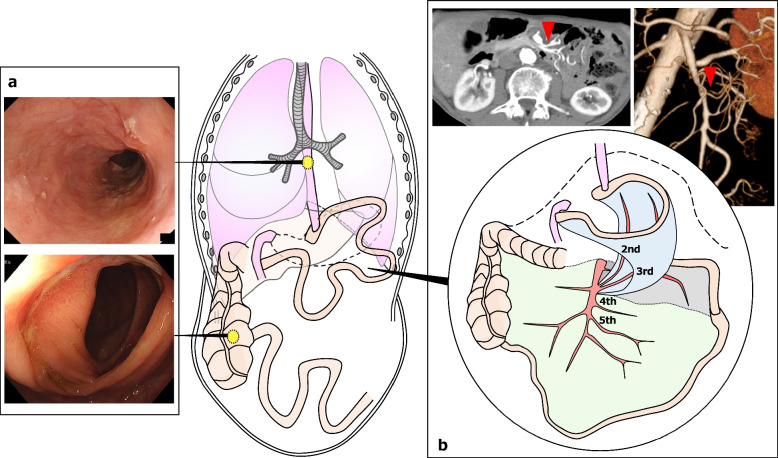
Findings after neoadjuvant chemotherapy and schema of the reconstruction methods. **a** Both esophageal and cecal cancers showed partial response. **b** Contrast-enhanced computed tomography revealed that the method of reconstruction after total gastrectomy was a long-distance jejunal interposition using the dominant areas of the second and third jejunal vessels (red arrowheads)

A 71-year-old man was referred from another hospital with a complaint of difficulty in swallowing food. He was diagnosed as having stage III (T3, N1, M0) esophageal squamous cell carcinoma in the middle thoracic esophagus, according to the TNM classification, 8th edition (Fig. 1a). A lower gastrointestinal endoscopy performed for screening purposes indicated synchronous stage IIA (T3, N0, M0) cecal cancer (Fig. 1b). This patient had undergone a total gastrectomy for gastric cancer at another hospital 27 years earlier. Details of the surgical procedure, including the method of reconstruction and the stage of the disease, were unknown (Fig. 1c). Because esophageal cancer was at a higher stage than cecal cancer and the standard treatment for advanced esophageal cancer is preoperative chemotherapy [19], we first chose neoadjuvant chemotherapy (DCF: docetaxel 35 mg/m^2^, cisplatin 40 mg/m^2^, fluorouracil 400 mg/m^2^) as preoperative treatment [20, 21]. At that time, we decided to perform lower gastrointestinal endoscopy after each course to confirm that the cecal cancer was not worsening. After two courses of neoadjuvant chemotherapy, the treatment efficacy was determined to be a partial response for both the esophageal cancer (76% reduction) and the cecal cancer (87% reduction), based on Response Evaluation Criteria in Solid Tumors Criteria version 1.1 (Fig. 2a) [22]. Contrast-enhanced computed tomography and upper gastrointestinal angiography during the preoperative treatment revealed that the method of reconstruction after total gastrectomy was a relatively long-distance jejunal interposition using the dominant areas of the second and third jejunal vessels (Fig. 2b). The oral side of the interpositioned jejunum had an end-to-side anastomosis with the esophagus and the aboral side with the second part of the duodenum, respectively.

**Fig. 3:**
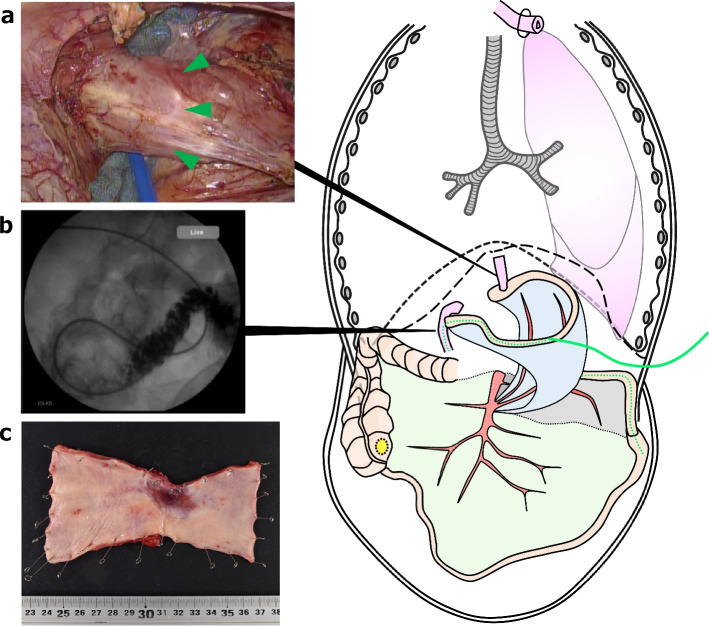
Intraoperative findings and schematic illustration in the first-stage operation. **a** The esophageal jejunal anastomotic line (green arrowheads) was identified in the thoracic cavity. **b** A jejunostomy (green dotted line) was created in the reconstructed jejunum, and the tip was placed at the third position of the duodenum. **c** Surgical specimen of the esophagus

**Fig. 4:**
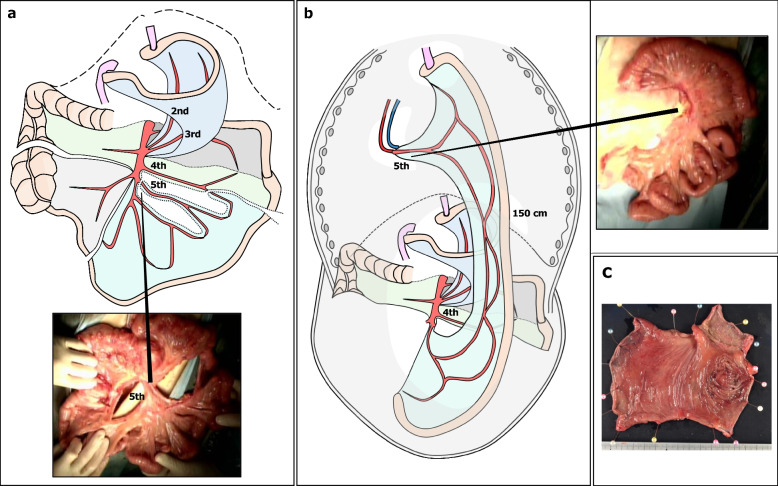
Intraoperative photograph and surgical schematic illustrations in the second-stage operation. **a** The left branch of the 4th jejunal artery was used as a feeder to the afferent loop, and the mesentery was incised to extend the elevation distance. **b** The jejunum was elevated approximately 150 cm by the presternal route, and the 5th jejunal artery and right internal thoracic artery were vaso-anastomosed. **c** Surgical specimen of the cecum

**Fig. 5:**
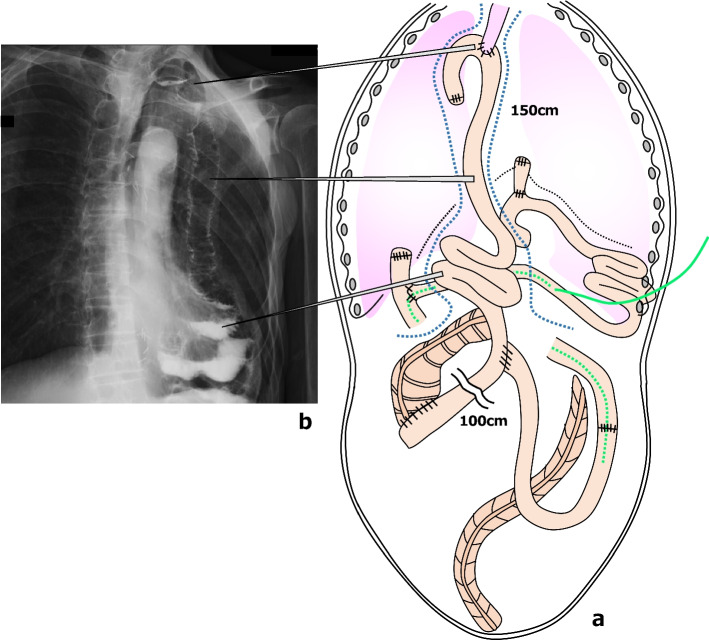
Schematic illustration of the final reconstruction with a subcutaneous tunnel (blue dotted line). **a** Postoperative upper gastrointestinal angiography.** b** Scheme of all anastomoses and blind ends

We planned esophagectomy as the first stage of the operation and ileocecal resection and gastrointestinal reconstruction as the second stage of the operation. In the first operation, the patient underwent thoracoscopic subtotal esophagectomy, two-field lymphadenectomy, cervical esophagostomy, and jejunostomy with a small laparotomy (Fig. 3). The esophageal cancer had invaded the thoracic duct and required combined resection. The adhesions to the crus were removed and the esophageal jejunal anastomosis was identified in the thoracic cavity, so the esophagus was dissected at the same site. With a small laparotomy, the jejunostomy was inserted through the reconstructed jejunum, and the tip was placed beyond Treitz’s ligament. The operative time was 7 h 15 min and blood loss was 30 mL. The postoperative course was generally uneventful, and the patient was discharged home on the 21st postoperative day. Six weeks after the first-stage operation, the second stage was performed. An ileocecal resection with D3 lymphadenectomy was performed first, followed by jejunal reconstruction. Considering the blood flow in the afferent loop including the past anastomosis, the left branch of the fourth jejunal artery was used as a feeder to the afferent loop, the right branch was included on the side of the elevated jejunum, and the mesentery was incised as long as possible to extend the elevation distance (Fig. 4a). A subcutaneous tunnel was created, the jejunum was elevated approximately 150 cm by the presternal route, and the fifth jejunal vein and the right internal thoracic vein were first anastomosed, and then the fifth jejunal artery and the right internal thoracic artery were anastomosed (Fig. 4b). Eight weeks had passed since the end of preoperative chemotherapy, but there was no progression of cecal cancer (Fig. 4c). After additional resection of the cervical esophagus, an end-to-side anastomosis was performed between the esophagus and the elevated jejunum, and a functional end-to-end anastomosis between the ascending colon and the elevated jejunum (Fig. 5a). The operative time was 9 h 55 min and blood loss was 410 mL. Because of many anastomoses and blind ends were created after a total of three operations (Fig. 5b), the patient developed mild leakage at the esophageal jejunal anastomosis postoperatively, which quickly resolved with conservative treatment. The esophageal cancer was in final stage I (T1a, N0 [0/17], M0, Grade 2) and the cecum cancer was also in final stage I (T2, N0 [0/15], M0, Grade 2), both of which could be curatively resected. At 15 months after surgery, the patient remains recurrence free from both cancers. Postoperatively, nutritional support using the jejunostomy was continued for 3 months, and the jejunostomy fistula was removed because the patient was able to take adequate oral intake.

## Discussion and conclusions

In patients with multiple cancers combined with esophageal cancer, a highly curative treatment such as that in the present case may be provided by choosing a staged operation, if necessary, in view of the operation time and degree of invasiveness. To achieve a better therapeutic effect, careful preoperative surgical planning is necessary, along with a multidisciplinary treatment plan that includes high-intensity preoperative chemotherapy and nutritional therapy [23]. We could find no report of treatment strategies for the simultaneous development of advanced thoracic esophageal and cecal cancers after total gastrectomy in our search of PubMed, and thus, we consider this to be the first reported case of multidisciplinary treatment strategies including anticancer drugs and surgical techniques for such a case.

In addition to the concept of field cancerization [6], it has been reported that a history of gastrectomy is associated with the development of lower esophageal squamous cell carcinoma due to duodenal gastroesophageal reflux [24, 25], and not a few cases are encountered that require esophagectomy after gastrectomy. Patients with thoracic esophageal cancer frequently have a history of gastrectomy that ranges from 2.8–10.4% [25–27] among all patients with esophageal cancer who underwent surgery. Surgical treatment of esophageal cancer patients after gastrectomy is more complex than conventional esophagectomy but is tolerable and should be considered as a reliable therapeutic modality because of the favorable patient prognosis [28].

It is necessary to have a thorough understanding of the advantages and disadvantages of the reconstructive methods for esophageal cancer cases after gastrectomy. In general, colon graft interposition and pedicled jejunum flap are often chosen for esophageal reconstruction in patients with a history of gastrectomy or those who have undergone synchronous esophagogastrostomy. Although colon graft interposition was selected for 60% of these patients in the late 1980s, the use of the pedicled jejunal flap has been gradually increasing and has recently reached more than 50% [29]. Two main types of colon graft interposition are traditionally used for esophageal reconstruction. The first is a right colon graft that uses the ileocecal or middle colic vessels as pedicles, and a segment from the terminal ileum to the ascending colon is interposed isoperistaltically. The other is a left colon graft that uses the ascending branch of the left colic artery and the inferior mesenteric vein as pedicles, and a segment from the transverse colon to the splenic flexure is used for interposition, also in an isoperistaltic fashion. Because of its multiple advantages such as Bauhin’s valve preventing regurgitation after reconstruction, the reservoir-like capacity of the cecum, and the close match in the diameter of the esophagus and ileum, the use of a right colon graft is usually recommended. However, the development of colon cancer after reconstruction is also an important problem [30, 31]. The jejunum has been used as an alternative conduit, both as a pedicled or free flap interposition with microvascular anastomosis. While a pedicle jejunum flap offers advantages such as fewer anastomoses, rare malignancy, and vigorous peristalsis, it also has disadvantages such as no reservoir and possible ischemia or congestion. As well, it is sometimes difficult to create a pedicle jejunum flap of sufficient length, especially in obese patients with thick mesentery, and elevation is often dictated by the length of the mesentery. Doki et al. performed a retrospective study to compare the peri-operative and long-term results of these two procedures [32] and reported no difference in operating time and blood loss. Compared with the colon reconstruction group, the hospital stay of the jejunum reconstruction group was significantly shorter and the incidence of anastomotic leakage tended to be less, whereas other operative morbidity did not differ between the two procedures. Bodyweight loss was less in the jejunum reconstruction group than in the colon group and showed a significant difference at 12 months after surgery. Supercharged vascular anastomosis is an important procedure that can reestablish or maintain gastrointestinal continuity in high-risk patients when the stomach is unavailable [33].

There are no previous reports of surgery for synchronous cancers of the esophagus and cecum after total gastrectomy with reconstruction by jejunal interposition. Although this case is extremely rare, it may be encountered in practice because some reports suggest that jejunal interposition is better than Roux-en-Y reconstruction for total gastrectomy [34]. The decision on surgical technique in this patient was very difficult, and we planned a two-stage operation after a thorough preoperative review. Although one-step operation is preferable considering the invasiveness of the procedure to the patient, we chose for two-stage operation in this case because our patient was in a post-total gastrectomy state and the operation was performed after preoperative chemotherapy which is the standard treatment for esophageal cancer in Japan. Three surgeries were necessary in this case: esophagectomy, ileocecal resection, and reconstruction using the small intestine. Key points of these surgeries are that reconstruction after total gastrectomy requires a relatively long-distance jejunal interposition using the area dominated by the second and third jejunal vessels, that the jejunum to be raised for reconstruction is defined by the length of the mesentery, and that there is concern about short bowel syndrome. In the present case, we chose to perform a two-stage operation because of the high degree of adhesions in the abdominal cavity and the long operation time that would be expected if all of the procedures, including the vascular anastomosis, were performed. Anastomosis of the aboral side of the elevated jejunum to the previously interposed jejunum might have been a better approach. Also, the interposed jejunum of the blind loop might have been better resected. However, since neither anastomosis nor resection was possible due to the high degree of adhesions, we chose the method presented in favor of safety. Oncologically, it might have been better to perform esophagectomy and ileocecal resection in the initial surgery. However, if the ileocecal resection were performed in the first stage of surgery, the second stage of surgery would be the third abdominal surgery this patient, which might have a negative impact on jejunal elevation. In terms of multidisciplinary treatment, because preoperative chemotherapy had also achieved a near complete response to the cecal cancer, we decided to separately perform the thoracic and abdominal operations. We consider that cisplatin [35] and fluorouracil [36], which are used in the standard treatment of advanced esophageal cancer, were also highly effective in treating the cecal cancer, which contributed to the good results. However, since there is no evidence of preoperative chemotherapy for colorectal cancer, lower gastrointestinal endoscopy was performed after each course of DCF to confirm that the cecal cancer had not progressed.

In conclusion, the procedure reported here may be recommended as an option for staged resection and reconstruction in patients with simultaneous advanced esophageal and cecal cancer after total gastrectomy.

## Data Availability

No datasets were generated or analysed during the current study.

## Abbreviation

DCF: Docetaxel cisplatin fluorouracil
